# Author Correction: Low-intensity pulsed ultrasound stimulation (LIPUS) modulates microglial activation following intracortical microelectrode implantation

**DOI:** 10.1038/s41467-024-52088-w

**Published:** 2024-09-02

**Authors:** Fan Li, Jazlyn Gallego, Natasha N. Tirko, Jenna Greaser, Derek Bashe, Rudra Patel, Eric Shaker, Grace E. Van Valkenburg, Alanoud S. Alsubhi, Steven Wellman, Vanshika Singh, Camila Garcia Padilla, Kyle W. Gheres, John I. Broussard, Roger Bagwell, Maureen Mulvihill, Takashi D. Y. Kozai

**Affiliations:** 1 Department of Bioengineering, University of Pittsburgh, Pittsburgh, PA USA; 2 Center for Neural Basis of Cognition, Pittsburgh, PA USA; 3 Computational Modeling and Simulation PhD Program, University of Pittsburgh, Pittsburgh, PA USA; 4 Department of Biochemistry and Molecular Biology, Pennsylvania State University, University Park, PA USA; 5 Actuated Medical, Bellefonte, PA USA; 6 Washington University in St. Louis, St. Louis, MO USA; 7 Department of Neuroscience, University of Pittsburgh, Pittsburgh, PA USA; 8 Columbia University, New York, NY USA; 9 Center for Neuroscience, University of Pittsburgh, Pittsburgh, PA USA; 10 McGowan Institute of Regenerative Medicine, University of Pittsburgh, Pittsburgh, PA USA; 11 NeuroTech Center, University of Pittsburgh Brain Institute, Pittsburgh, PA USA

**Keywords:** Implants, Microglia, Neurodegenerative diseases

Correction to: *Nature Communications* 10.1038/s41467-024-49709-9, published online 29 June 2024

The original version of this Article contained an error in Fig. 8, in which panels d, e had the *y* axis units incorrectly formatted (..V), panel f had the wrong *y*-axis unit (mOhms), and panel g had the *x*-axis units incorrectly formatted (..m).

The correct version of Fig. 8 is:
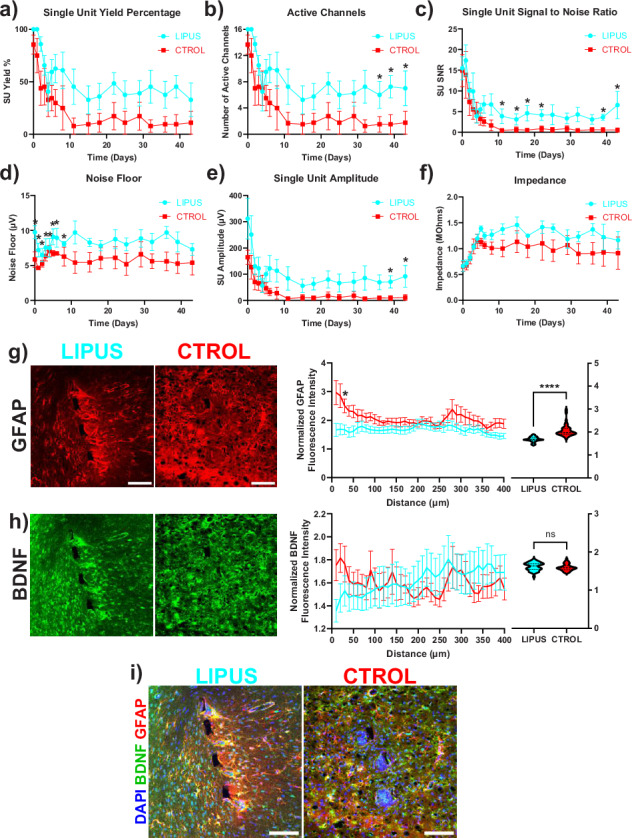


which replaces the previous incorrect version:
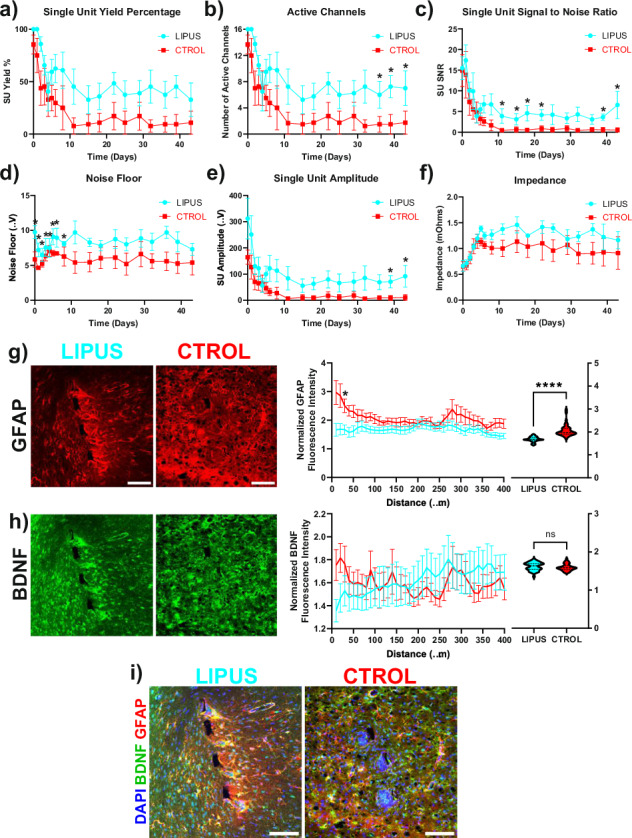


This has been corrected in both the PDF and HTML versions of the Article.

