# Supplementary material for: Low-intensity pulsed ultrasound stimulation (LIPUS) modulates microglial activation following intracortical microelectrode implantation
Source: Nat Commun. 2024 Jun 29;15:5512. doi: 10.1038/s41467-024-49709-9 (PMC11217463; doi:10.1038/s41467-024-49709-9)
Supplement: Supplementary file 1 — Supplementary Information [file 41467_2024_49709_MOESM1_ESM.pdf]

Low-intensity pulsed ultrasound stimulation (LIPUS) modulates microglial activation following intracortical microelectrode implantation.

Fan Li#[1,2,3], Jazlyn Gallego#[1,2], Natasha N Tirko[4], Jenna Greaser[5], Derek Bashe[6], Rudra Patel[7], Eric Shaker[1], Grace E Van Valkenburg[1], Alanoud S Alsubhi[5], Steven Wellman[8], Vanshika Singh[1], Camila Garcia Padilla[1,2], Kyle W. Gheres[5], John I. Broussard[5], Roger Bagwell[5], Maureen Mulvihill[5], Takashi D.Y. Kozai[1,2,9-11*]

#Denotes equal contribution
* Corresponding author: TK.Kozai@pitt.edu
1Department of Bioengineering, University of Pittsburgh, Pittsburgh, PA, USA
2Center for Neural Basis of Cognition, Pittsburgh, PA, USA
3Computational Modeling and Simulation PhD program, University of Pittsburgh, Pittsburgh, PA, USA
4Department of Biochemistry and Molecular Biology, Pennsylvania State University, University Park, PA, USA
5Actuated Medical, Bellefonte, PA, USA
6Washington University in St. Louis, St. Louis, MO, USA
7Department of Neuroscience, University of Pittsburgh, Pittsburgh, PA, USA
8Columbia University, New York, NY, USA
9Center for Neuroscience, University of Pittsburgh, Pittsburgh, PA, USA
10McGowan Institute of Regenerative Medicine, University of Pittsburgh, Pittsburgh, PA, USA
11NeuroTech Center, University of Pittsburgh Brain Institute, Pittsburgh, PA, USA

Supplementary Materials

Supplementary tables

Table 1: N and n for velocity of migrating microglia analysis (Figure 2)

Day	LIPUS N	Control N	LIPUS n	Control n
1	6	5	36	29
2	7	5	41	28
3	7	4	40	23
4	7	4	38	22
5	7	4	37	23
6	5	4	23	22
7	2	4	7	17

N = number of animals
n = number of cells

Table 2: N and n for microglial activation analysis (Figure 3)

Bin			0-50		50-100		100-150		150-200		200-250		250-300	
Day	LIPUS N	Control N	LIPUS n	Control n	LIPUS n	Control n	LIPUS n	Control n	LIPUS n	Control n	LIPUS n	Control n	LIPUS n	Control n
1	6	6	6	4	10	16	5	12	16	15	16	8	7	9
2	6	5	4	10	16	10	14	13	19	10	16	7	10	10
3	5	5	5	9	11	9	10	9	11	9	12	11	8	11
4	6	4	7	5	3	4	10	7	14	10	13	8	12	13
5	5	5	17	11	27	23	26	14	18	17	17	12	8	10
6	5	4	21	40	29	29	23	23	27	25	24	23	19	23
7	4	5	16	8	24	17	17	13	14	16	6	14	5	14

N = number of animals
n = number of cells

Table 3: N and n for microglia surveillance analysis (Figure 4)

Day	LIPUS N	Control N	LIPUS n	Control n
0	3	3	14	16
1	4	3	17	14
2	3	4	26	17
3	3	3	13	15
4	3	3	15	15
5	2	2	12	14
6	4	3	10	11
7	4	2	16	9

N = number of animals
n = number of cells

Table 4: N and n for microglial encapsulation analysis (Figure 5)

Day	LIPUS N	Control N
0	7	6
1	7	6
2	7	6
3	7	6
4	7	6
5	7	6
6	7	6
7	7	6
14	4	6
21	3	5
28	3	4

N = number of animals
n = number of cells

Table 5: N and n for vessel-associated microglia analysis (Figure 6)

Day	LIPUS N	Control N
1	7	5
2	6	6
3	6	6
4	7	6
5	7	5
6	7	6
7	6	6

N = number of animals
n = number of cells

Table 6: N and n for blood vessel analysis (Figure 7)

Day	LIPUS N	Control N	LIPUS n	Control n
0	3	4	7	5
1	3	4	7	5
2	3	4	7	5
3	3	4	7	5
4	3	4	7	5
5	3	4	7	5
6	3	4	7	5
7	3	4	7	5
14	3	4	7	5
21	3	4	7	5
28	3	4	7	5

N = number of animals
n = number of cells

Table 7: N and n for electrophysiology and immunohistochemistry analysis (Figure 8)

	Rats		Probe Shank	
	LIPUS N	Control N	LIPUS n	Control n
Ephys (d0-43)	4	4	10	9
GFAP (d43)	3	3	10	9
BDNF(d43)	3	3	10	9

N = number of animals
n = number of probe shanks quantified
