# Supplementary material for: Low-intensity pulsed ultrasound stimulation (LIPUS) modulates microglial activation following intracortical microelectrode implantation
Source: Nat Commun. 2024 Jun 29;15:5512. doi: 10.1038/s41467-024-49709-9 (PMC11217463; doi:10.1038/s41467-024-49709-9)
Supplement: Supplementary file 3 — Reporting summary [file 41467_2024_49709_MOESM3_ESM.pdf]

nature portfolio

Corresponding author(s): Takashi Kozai
Last updated by author(s): May 24, 2024

Reporting Summary

Nature Portfolio wishes to improve the reproducibility of the work that we publish. This form provides structure for consistency and transparency in reporting. For further information on Nature Portfolio policies, see our Editorial Policies and the Editorial Policy Checklist.

Statistics

For all statistical analyses, confirm that the following items are present in the figure legend, table legend, main text, or Methods section.

n/a	Confirmed	
☐	☒	The exact sample size (*n*) for each experimental group/condition, given as a discrete number and unit of measurement
☐	☒	A statement on whether measurements were taken from distinct samples or whether the same sample was measured repeatedly
☐	☒	The statistical test(s) used AND whether they are one- or two-sided<br>*Only common tests should be described solely by name; describe more complex techniques in the Methods section.*
☐	☒	A description of all covariates tested
☐	☒	A description of any assumptions or corrections, such as tests of normality and adjustment for multiple comparisons
☐	☒	A full description of the statistical parameters including central tendency (e.g. means) or other basic estimates (e.g. regression coefficient) AND variation (e.g. standard deviation) or associated estimates of uncertainty (e.g. confidence intervals)
☐	☒	For null hypothesis testing, the test statistic (e.g. *F*, *t*, *r*) with confidence intervals, effect sizes, degrees of freedom and *P* value noted<br>*Give P values as exact values whenever suitable.*
☒	☐	For Bayesian analysis, information on the choice of priors and Markov chain Monte Carlo settings
☒	☐	For hierarchical and complex designs, identification of the appropriate level for tests and full reporting of outcomes
☒	☐	Estimates of effect sizes (e.g. Cohen's *d*, Pearson's *r*), indicating how they were calculated

Our web collection on statistics for biologists contains articles on many of the points above.

Software and code

Policy information about availability of computer code

Data collection	ImageJ
Data analysis	ImageJ, MATLAB, Python

For manuscripts utilizing custom algorithms or software that are central to the research but not yet described in published literature, software must be made available to editors and reviewers. We strongly encourage code deposition in a community repository (e.g. GitHub). See the Nature Portfolio guidelines for submitting code & software for further information.

Data

Policy information about availability of data

All manuscripts must include a data availability statement. This statement should provide the following information, where applicable:

- Accession codes, unique identifiers, or web links for publicly available datasets
- A description of any restrictions on data availability
- For clinical datasets or third party data, please ensure that the statement adheres to our policy

The data that support the findings of this study are available from the corresponding author upon reasonable request.

Research involving human participants, their data, or biological material

Policy information about studies with human participants or human data. See also policy information about sex, gender (identity/presentation), and sexual orientation and race, ethnicity and racism.

Reporting on sex and gender	Research did not involve human participants
Reporting on race, ethnicity, or other socially relevant groupings	Research did not involve human participants
Population characteristics	Research did not involve human participants
Recruitment	Research did not involve human participants
Ethics oversight	Research did not involve human participants

Note that full information on the approval of the study protocol must also be provided in the manuscript.

Field-specific reporting

Please select the one below that is the best fit for your research. If you are not sure, read the appropriate sections before making your selection.

☒ Life sciences ☐ Behavioural & social sciences ☐ Ecological, evolutionary & environmental sciences

For a reference copy of the document with all sections, see nature.com/documents/nr-reporting-summary-flat.pdf

Life sciences study design

All studies must disclose on these points even when the disclosure is negative.

Sample size	The sample size was determined using a power analysis of previous microglia-GFP experiments.
Data exclusions	Data were excluded if the imaging window precluded the ability to quantify individual cells or if the animal's headcap was inadvertently removed.
Replication	All quantifiable replicates were included in the presented data. The data were successfully replicated.
Randomization	Surgeries were conducted in pairs so that one was designated experimental group and the other designated control. Surgeon was blinded to which group the animals would be assigned to by the post-op team.
Blinding	The surgeon was blinded to which group each animal will belong to. Analyses were repeated by multiple individuals unblinded.

Reporting for specific materials, systems and methods

We require information from authors about some types of materials, experimental systems and methods used in many studies. Here, indicate whether each material, system or method listed is relevant to your study. If you are not sure if a list item applies to your research, read the appropriate section before selecting a response.

Materials & experimental systems

n/a	Involved in the study	
☐	☒	Antibodies
☒	☐	Eukaryotic cell lines
☒	☐	Palaeontology and archaeology
☐	☒	Animals and other organisms
☒	☐	Clinical data
☒	☐	Dual use research of concern
☒	☐	Plants

Methods

n/a	Involved in the study	
☒	☐	ChIP-seq
☒	☐	Flow cytometry
☒	☐	MRI-based neuroimaging

Antibodies

Antibodies used	Brain Derived Neurotrophic Factor (Abcam, Recombinant Anti-BDNF antibody [EPR1292], catalog #ab108319, Lot #GR3227037-10 (0.271mg/ml) ,clonality: monoclonal, 1:100 dilution), Glial Fibrillary Acidic Protein (Millipore Sigma, Monoclonal Anti-Glial Fibrillary Acidic Protein, catalog #G383, Lot #0000122915 (4-8mg/mL), clone: G-A-5, clonality: monoclonal, 1:250 dilution), Alexa Fluor 488

secondary antibody (Abcam, Donkey Anti-Rabbit IgG H&L (Alexa Fluor® 488), catalog #ab150073, Lot # GR3313306-1 (2.00mg/ml), clonality: polyclonal, 1:250 dilution), Alexa Fluor 594 secondary antibody (Abcam, Donkey anti-goat IgG H&L Alexa Fluor 594, catalog #ab150132, Lot #GR3290061-3 (2.00mg/ml), clonality: polyclonal, 1:500 dilution)

Validation

ab108319 has been referenced in 380 publications, most recently:

Wang WZ et al. Diffusion tensor imaging of the hippocampus reflects the severity of hippocampal injury induced by global cerebral ischemia/reperfusion injury. Neural Regen Res 17:838-844 (2022). PubMed: 34472484
Cramer T et al. Cross-talk between GABAergic postsynapse and microglia regulate synapse loss after brain ischemia. Sci Adv 8:eabj0112 (2022). PubMed: 35245123
Wang S et al. BDNF and TrKB expression levels in patients with endometriosis and their associations with dysmenorrhoea. J Ovarian Res 15:35 (2022). PubMed: 35300713
Fu Y et al. Loss of neurodevelopmental-associated miR-592 impairs neurogenesis and causes social interaction deficits. Cell Death Dis 13:292 (2022). PubMed: 35365601
Xue Z et al. Role of BDNF/ProBDNF Imbalance in Postoperative Cognitive Dysfunction by Modulating Synaptic Plasticity in Aged Mice. Front Aging Neurosci 14:780972 (2022). PubMed: 35370607
Lin L et al. The role and mechanism of TLR4-siRNA in the impairment of learning and memory in young mice induced by isoflurane. Adv Clin Exp Med 31:769-780 (2022). PubMed: 35394127
Wen J et al. The cAMP Response Element- Binding Protein/Brain-Derived Neurotrophic Factor Pathway in Anterior Cingulate Cortex Regulates Neuropathic Pain and Anxiodepression Like Behaviors in Rats. Front Mol Neurosci 15:831151 (2022). PubMed: 35401106
Jhan KY et al. Synaptic loss and progression in mice infected with Angiostrongylus cantonensis in the early stage. J Neuroinflammation 19:85 (2022). PubMed: 35414007
Yuan HJ et al. Glucocorticoid Exposure of Preimplantation Embryos Increases Offspring Anxiety-Like Behavior by Upregulating miR-211-5p via Trpm1 Demethylation. Front Cell Dev Biol 10:874374 (2022). PubMed: 35433692
Chai Y et al. Salidroside Ameliorates Depression by Suppressing NLRP3-Mediated Pyroptosis via P2X7/NF-κB/NLRP3 Signaling Pathway. Front Pharmacol 13:812362 (2022). PubMed: 35496273
Wang L et al. Overexpression of BDNF in the ventrolateral periaqueductal gray regulates the behavior of epilepsy-migraine comorbid rats. Brain Behav 12:e2594 (2022). PubMed: 35557046
Cao Q et al. Regulation of BDNF transcription by Nrf2 and MeCP2 ameliorates MPTP-induced neurotoxicity. Cell Death Discov 8:267 (2022). PubMed: 35595779
George SD et al. Caloric Vestibular Stimulation Induced Enhancement of Behavior and Neurotrophic Factors in Chronic Mild Stress Induced Rats. Front Pharmacol 13:834292 (2022). PubMed: 35600855
Fang Z et al. Blueberry Anthocyanins Extract Attenuates Acrylamide-Induced Oxidative Stress and Neuroinflammation in Rats. Oxid Med Cell Longev 2022:7340881 (2022). PubMed: 35651724
Peng D et al. Extracellular vesicles derived from astrocyte-treated with haFGF14-154 attenuate Alzheimer phenotype in AD mice. Theranostics 12:3862-3881 (2022). PubMed: 35664060
Zhang Z et al. Hippocampal Mitochondrial Transplantation Alleviates Age-Associated Cognitive Decline via Enhancing Wnt Signaling and Neurogenesis. Comput Intell Neurosci 2022:9325302 (2022). PubMed: 35685133
Zhang Q et al. Implantation of a nerve protector embedded with human GMSC-derived Schwann-like cells accelerates regeneration of crush-injured rat sciatic nerves. Stem Cell Res Ther 13:263 (2022). PubMed: 35725660
Yang Y et al. Constant light in early life induces fear-related behavior in chickens with suppressed melatonin secretion and disrupted hippocampal expression of clock- and BDNF-associated genes. J Anim Sci Biotechnol 13:67 (2022). PubMed: 35729672
Fu L et al. Dietary Supplement of Anoectochilus roxburghii (Wall.) Lindl. Polysaccharides Ameliorates Cognitive Dysfunction Induced by High Fat Diet via "Gut-Brain" Axis. Drug Des Devel Ther 16:1931-1945 (2022). PubMed: 35762015
Gao J et al. Panax notoginseng Saponins Stimulates Neurogenesis and Neurological Restoration After Microsphere-Induced Cerebral Embolism in Rats Partially Via mTOR Signaling. Front Pharmacol 13:889404 (2022).

GG383 has been referenced in 1322 publications including
1-30 of 1322 results for "G3893" within Papers
A three-dimensional model of the human blood-brain barrier to analyse the transport of nanoparticles and astrocyte/endothelial interactions.
Peddagangannagari Sreekanthreddy et al.
F1000Research, 4, 1279-1279 (2016-02-13)
The aim of this study was to develop a three-dimensional (3D) model of the human blood-brain barrier in vitro, which mimics the cellular architecture of the CNS and could be used to analyse the delivery of nanoparticles to cells of
Effects of ischemia-reperfusion on physiological properties of Müller glial cells in the porcine retina.
Antje Wurm et al.
Investigative ophthalmology & visual science, 52(6), 3360-3367 (2011-02-25)
Transient retinal ischemia-reperfusion is associated with neuronal degeneration and activation of Müller glial cells. Reactive gliosis may impede the homeostatic functions of Müller cells. A viable animal model for human ischemic events should display similarities in eye size and retinal
Wnts are expressed in the spinal cord of adult mice and are differentially induced after injury.
Carlos González-Fernández et al.
Journal of neurotrauma, 31(6), 565-581 (2013-12-26)
The Wnt family of proteins plays key roles during central nervous system development and has been involved in several neuropathologies during adulthood, including spinal cord injury (SCI). However, Wnts expression knowledge is relatively limited during adult stages. Here, we sought
Dentate gyrus morphogenesis is regulated by β-catenin function in hem-derived fimbrial glia.
Arpan Parichha et al.
Development (Cambridge, England), 149(21) (2022-10-06)
The dentate gyrus, a gateway for input to the hippocampal formation, arises from progenitors in the medial telencephalic neuroepithelium adjacent to the cortical hem. Dentate progenitors navigate a complex migratory path guided by two cell populations that arise from the
Citron kinase is required for postnatal neurogenesis in the hippocampus.
James B Ackman et al.
Developmental neuroscience, 29(1-2), 113-123 (2006-12-07)

The dentate gyrus is a site of continual neurogenesis in the postnatal mammalian brain. Here we investigated postnatal neurogenesis in the citron kinase (citron-K) null-mutant rat (flathead). The flathead rat has substantial deficits in embryonic neurogenesis that are due to
A seasonal switch in histone deacetylase gene expression in the hypothalamus and their capacity to modulate nuclear signaling pathways.
Patrick N Stoney et al.
Brain, behavior, and immunity, 61, 340-352 (2016-12-21)
Seasonal animals undergo changes in physiology and behavior between summer and winter conditions. These changes are in part driven by a switch in a series of hypothalamic genes under transcriptional control by hormones and, of recent interest, inflammatory factors. Crucial
Divergent single cell transcriptome and epigenome alterations in ALS and FTD patients with C9orf72 mutation.
Junhao Li et al.
Nature communications, 14(1), 5714-5714 (2023-09-16)
A repeat expansion in the C9orf72 (C9) gene is the most common genetic cause of amyotrophic lateral sclerosis (ALS) and frontotemporal dementia (FTD). Here we investigate single nucleus transcriptomics (snRNA-seq) and epigenomics (snATAC-seq) in postmortem motor and frontal cortices from
Surface topography during neural stem cell differentiation regulates cell migration and cell morphology.
Catherine Czeisler et al.
The Journal of comparative neurology, 524(17), 3485-3502 (2016-07-16)
We sought to determine the contribution of scaffold topography to the migration and morphology of neural stem cells by mimicking anatomical features of scaffolds found in vivo. We mimicked two types of central nervous system scaffolds encountered by neural stem
NG2 glia regulate brain innate immunity via TGF-β2/TGFBR2 axis.
Shu-Zhen Zhang et al.
BMC medicine, 17(1), 204-204 (2019-11-16)
Brain innate immunity is vital for maintaining normal brain functions. Immune homeostatic imbalances play pivotal roles in the pathogenesis of neurological diseases including Parkinson's disease (PD). However, the molecular and cellular mechanisms underlying the regulation of brain innate immunity and
Opposite effects of a high-fat diet and calorie restriction on ciliary neurotrophic factor signaling in the mouse hypothalamus.
Ilenia Severi et al.
Frontiers in neuroscience, 7, 263-263 (2014-01-11)
In the mouse hypothalamus, ciliary neurotrophic factor (CNTF) is mainly expressed by ependymal cells and tanycytes of the ependymal layer covering the third ventricle. Since exogenously administered CNTF causes reduced food intake and weight loss, we tested whether endogenous CNTF
Donepezil promotes differentiation of neural stem cells into mature oligodendrocytes at the expense of astrogenesis.
Osamu Imamura et al.
Journal of neurochemistry, 140(2), 231-244 (2016-10-19)
Oligodendrocytes are the myelin-forming cells of the central nervous system. Oligodendrocyte loss and failure of myelin development result in serious human disorders, including multiple sclerosis. Previously, using oligodendrocyte progenitor cells, we have shown that donepezil, which is an acetylcholinesterase inhibitor
Heterogeneity of astrocytes: Electrophysiological properties of juxtavascular astrocytes before and after brain injury.
Stefanie Götz et al.
Glia, 69(2), 346-361 (2020-08-19)
Astrocyte heterogeneity is increasingly recognized, but still little is known about juxtavascular astrocytes with their somata directly adjacent to blood vessels, despite their importance after brain injury. As juxtavascular astrocytes originate from common progenitor cells, that is, have a clonal
Effects of sleep and wake on astrocytes: clues from molecular and ultrastructural studies.
Michele Bellesi et al.
BMC biology, 13, 66-66 (2015-08-26)
Astrocytes can mediate neurovascular coupling, modulate neuronal excitability, and promote synaptic maturation and remodeling. All these functions are likely to be modulated by the sleep/wake cycle, because brain metabolism, neuronal activity and synaptic turnover change as a function of behavioral
Dynamic expression patterns of G protein-regulated inducer of neurite outgrowth 1 (GRIN1) and its colocalization with Galphao implicate significant roles of Galphao-GRIN1 signaling in nervous system.
Ikuo Masuho et al.
Developmental dynamics : an official publication of the American Association of Anatomists, 237(9), 2415-2429 (2008-08-30)
GRIN1 (Gprin 1) is a signaling molecule coexpression of which with constitutively active form of Galphao can stimulate neurite extensions in Neuro2a cells, yet its in vivo roles remain elusive. Here, we examine expression profiles of GRIN1 during mouse development
Reduction of the expression of the late-onset Alzheimer's disease (AD) risk-factor BIN1 does not affect amyloid pathology in an AD mouse model.
Robert J Andrew et al.
The Journal of biological chemistry, 294(12), 4477-4487 (2019-01-30)
Alzheimer's disease (AD) is pathologically characterized by the deposition of the β-amyloid (Aβ) peptide in senile plaques in the brain, leading to neuronal dysfunction and eventual decline in cognitive function. Genome-wide association studies have identified the bridging integrator 1 (BIN1)
Myeloperoxidase-immunoreactive cells are significantly increased in brain areas affected by neurodegeneration in Parkinson's and Alzheimer's disease.
Sandra Gellhaar et al.
Cell and tissue research, 369(3), 445-454 (2017-05-04)
Myeloperoxidase (MPO) is a key enzyme in inflammatory and degenerative processes, although conflicting reports have been presented concerning its expression in the brain. We studied the cellular localization of MPO and compared numbers of MPO cells in various brain regions
Plasticity of motor network and function in the absence of corticospinal projection.
Qi Han et al.
Experimental neurology, 267, 194-208 (2015-03-21)

Despite the obvious clinical interest, our understanding of how developmental mechanisms are redeployed during degeneration and regeneration after brain and spinal cord injuries remains quite rudimentary. In animal models of spinal cord injury, although spontaneous regeneration of descending axons is
Glucocorticoids Target Ependymal Glia and Inhibit Repair of the Injured Spinal Cord.
Craig M Nelson et al.
Frontiers in cell and developmental biology, 7, 56-56 (2019-05-10)
Following injury, the mammalian spinal cord forms a glial scar and fails to regenerate. In contrast, vertebrate fish spinal cord tissue regenerates significantly to restore function. Cord transection in zebrafish (Danio rerio) initially causes paralysis and neural cell death. Subsequently
Alborixin clears amyloid-β by inducing autophagy through PTEN-mediated inhibition of the AKT pathway.
Abubakar Wani et al.
Autophagy, 15(10), 1810-1828 (2019-03-22)
Imbalance in production and clearance of amyloid beta (Aβ) is the primary reason for its deposition in Alzheimer disease. Macroautophagy/autophagy is one of the important mechanisms for clearance of both intracellular and extracellular Aβ. Here, through screening, we identified alborixin
Mechanisms of NMDA Receptor- and Voltage-Gated L-Type Calcium Channel-Dependent Hippocampal LTP Critically Rely on Proteolysis That Is Mediated by Distinct Metalloproteinases.
Grzegorz Wiera et al.
The Journal of neuroscience : the official journal of the Society for Neuroscience, 37(5), 1240-1256 (2017-01-11)
Long-term potentiation (LTP) is widely perceived as a memory substrate and in the hippocampal CA3-CA1 pathway, distinct forms of LTP depend on NMDA receptors (nmdaLTP) or L-type voltage-gated calcium channels (vdccLTP). LTP is also known to be effectively regulated by
Microglial debris is cleared by astrocytes via C4b-facilitated phagocytosis and degraded via RUBICON-dependent noncanonical autophagy in mice.
Tian Zhou et al.
Nature communications, 13(1), 6233-6233 (2022-10-25)
Microglia are important immune cells in the central nervous system (CNS) that undergo turnover throughout the lifespan. If microglial debris is not removed in a timely manner, accumulated debris may influence CNS function. Clearance of microglial debris is crucial for
Differential temporal and spatial post-injury alterations in cerebral cell morphology and viability.
Zareen Amtul et al.
The Journal of comparative neurology, 529(2), 421-433 (2020-05-25)
Combination of ischemia and β-amyloid (Aβ) toxicity has been shown to simultaneously increase neuro-inflammation, endogenous Aβ deposition, and neurodegeneration. However, studies on the evolution of infarct and panorama of cellular degeneration as a synergistic or overlapping mechanism between ischemia and
In-vitro engineered human cerebral tissues mimic pathological circuit disturbances in 3D.
Aref Saberi et al.
Communications biology, 5(1), 254-254 (2022-03-25)
In-vitro modeling of brain network disorders such as epilepsy remains a major challenge. A critical step is to develop an experimental approach that enables recapitulation of in-vivo-like three-dimensional functional complexity while allowing local modulation of the neuronal networks. Here, by
Leptin increases sympathetic nerve activity via induction of its own receptor in the paraventricular nucleus.
Zhigang Shi et al.
eLife, 9 (2020-06-17)
Whether leptin acts in the paraventricular nucleus (PVN) to increase sympathetic nerve activity (SNA) is unclear, since PVN leptin receptors (LepR) are sparse. We show in rats that PVN leptin slowly increases SNA to muscle and brown adipose tissue, because
Inhibition of Dectin-1 Alleviates Neuroinflammatory Injury by Attenuating NLRP3 Inflammasome-Mediated Pyroptosis After Intracerebral Hemorrhage in Mice: Preliminary Study Results.
Zhiquan Ding et al.
Journal of inflammation research, 15, 5917-5933 (2022-10-25)
Neuroinflammation plays an important role following intracerebral hemorrhage (ICH). NLRP3 inflammasome-mediated pyroptosis contributes to the mechanism of neuroinflammation. It has been reported that dendritic cell-associated C-type lectin-1 (Dectin-1) activation triggers inflammation in neurological diseases. However, the role of Dectin-1 on
Budesonide with surfactant decreases systemic responses in mechanically ventilated preterm lambs exposed to fetal intra-amniotic lipopolysaccharide.
Noah H Hillman et al.
Pediatric research (2020-11-13)
Chorioamnionitis is associated with increased rates of bronchopulmonary dysplasia (BPD) in ventilated preterm infants. Budesonide when added to surfactant decreased lung and systemic inflammation from mechanical ventilation in preterm lambs and decreased the rates and severity of BPD in preterm
Repeated closed-head mild traumatic brain injury-induced inflammation is associated with nociceptive sensitization.
Tyler Nguyen et al.
Journal of neuroinflammation, 20(1), 196-196 (2023-08-28)
Individuals who have experienced mild traumatic brain injuries (mTBIs) suffer from several comorbidities, including chronic pain. Despite extensive studies investigating the underlying mechanisms of mTBI-associated chronic pain, the role of inflammation in long-term pain after mTBIs is not fully elucidated.
Dystrophin Distribution and Expression in Human and Experimental Temporal Lobe Epilepsy.
Ruben G F Hendriksen et al.
Frontiers in cellular neuroscience, 10, 174-174 (2016-07-28)
Dystrophin is part of a protein complex that connects the cytoskeleton to the extracellular matrix. In addition to its role in muscle tissue, it functions as an anchoring protein within the central nervous system such as in hippocampus and cerebellum.
Progesterone Attenuates Microglial-Driven Retinal Degeneration and Stimulates Protective Fractalkine-CX3CR1 Signaling.
Sarah L Roche et al.
PloS one, 11(11), e0165197-e0165197 (2016-11-05)
Retinitis pigmentosa (RP) is a degenerative disease leading to photoreceptor cell loss. Mouse models of RP, such as the rd10 mouse (B6.CXBl-Pde6brd10/J), have enhanced our understanding of the disease, allowing for development of potential therapeutics. In

2011, our group first
The expression of DJ-1 (PARK7) in normal human CNS and idiopathic Parkinson's disease.
Rina Bandopadhyay et al.
Brain : a journal of neurology, 127(Pt 2), 420-430 (2003-12-10)
Two mutations in the DJ-1 gene on chromosome1p36 have been identified recently to cause early-onset, autosomal recessive Parkinson's disease. As no information is available regarding the distribution of DJ-1 protein in the human brain, in this study we used

ab150077 has been used in 2871 publications most recently:
Lin Z et al. In situ immunomodulation of tumors with biosynthetic bacteria promote anti-tumor immunity. Bioact Mater 32:12-27 (2024). PubMed: 37790917
Fang Y et al. Human endogenous retroviruses as epigenetic therapeutic targets in TP53-mutated diffuse large B-cell lymphoma. Signal Transduct Target Ther 8:381 (2023). PubMed: 37798292
Fiock KL et al. Determinants of astrocytic pathology in stem cell models of primary tauopathies. Acta Neuropathol Commun 11:161 (2023). PubMed: 37803326
Liu TT et al. Endothelial cell-derived RSPO3 activates Gαi1/3-Erk signaling and protects neurons from ischemia/reperfusion injury. Cell Death Dis 14:654 (2023). PubMed: 37805583
Huynh QS & Holsinger RMD Fiber and Electrical Field Alignment Increases BDNF Expression in SH-SY5Y Cells following Electrical Stimulation. Pharmaceuticals (Basel) 16:N/A (2023). PubMed: 37259290
Zhang T et al. Daphnetin Improves Neuropathic Pain by Inhibiting the Expression of Chemokines and Inflammatory Factors in the Spinal Cord and Interfering with Glial Cell Polarization. Pharmaceuticals (Basel) 16:N/A (2023). PubMed: 37259390
Zhao T et al. Macrophages induce gingival destruction via Piezo1-mediated MMPs-degrading collagens in periodontitis. Front Immunol 14:1194662 (2023). PubMed: 37261355
Moya L et al. Characterisation of cell lines derived from prostate cancer patients with localised disease. Prostate Cancer Prostatic Dis 26:614-624 (2023). PubMed: 37264224
He N et al. HCFC1 variants in the proteolysis domain are associated with X-linked idiopathic partial epilepsy: Exploring the underlying mechanism. Clin Transl Med 13:e1289 (2023). PubMed: 37264743
Chen Y et al. CTHRC1 promotes anaplastic thyroid cancer progression by upregulating the proliferation, migration, and invasion of tumor cells. PeerJ 11:e15458 (2023). PubMed: 37273536
Yan H et al. iRHOM2 regulates inflammation and endothelial barrier permeability via CX3CL1. Exp Ther Med 26:319 (2023). PubMed: 37273752
Xiang J et al. Identification of DLL3-related genes affecting the prognosis of patients with colon adenocarcinoma. Front Genet 14:1098190 (2023). PubMed: 37274780
Matsukuma H et al. Prominin-1 deletion results in spermatogenic impairment, sperm morphological defects, and infertility in mice. Reprod Med Biol 22:e12514 (2023). PubMed: 37292088
Egorova TV et al. In-Frame Deletion of Dystrophin Exons 8-50 Results in DMD Phenotype. Int J Mol Sci 24:N/A (2023). PubMed: 37298068
Furukawa M et al. Long-Term Capsaicin Administration Ameliorates the Dysfunction and Astrogliosis of the Brain in Aged Mice with Missing Maxillary Molars. Nutrients 15:N/A (2023). PubMed: 37299434
Fang Z et al. Gasdermin D affects aortic vascular smooth muscle cell pyroptosis and Ang II-induced vascular remodeling. Heliyon 9:e16619 (2023). PubMed: 37303505
Svobodová Kovaříková A et al. PARP-dependent and NAT10-independent acetylation of N4-cytidine in RNA appears in UV-damaged chromatin. Epigenetics Chromatin 16:26 (2023). PubMed: 37322549
Gao XW et al. CX3CL1/CX3CR1 axis alleviates inflammation and apoptosis in human nucleus pulpous cells via M2 macrophage polarization. Exp Ther Med 26:359 (2023). PubMed: 37324510
Zhou M et al. Nanovesicles loaded with a TGF-β receptor 1 inhibitor overcome immune resistance to potentiate cancer immunotherapy. Nat Commun 14:3593 (2023). PubMed: 37328484
Li S et al. LSD1 interacting with HSP90 promotes skin wound healing by inducing metabolic reprogramming of hair follicle stem cells through the c-MYC/LDHA axis. FASEB J 37:e23031 (2023).

ab150132 has been used in 85 publications, most recently:
Yomogita H et al. A possible function of Nik-related kinase in the labyrinth layer of delayed delivery mouse placentas. J Reprod Dev 69:32-40 (2023). PubMed: 36567126
Yao M et al. Melatonin restores endoplasmic reticulum homeostasis to protect injured neurons in a rat model of chronic cervical cord compression. J Pineal Res 74:e12859 (2023). PubMed: 36732085
Zamith Cunha R et al. Expression of cannabinoid (CB1 and CB2) and cannabinoid-related receptors (TRPV1, GPR55, and PPARα) in the synovial membrane of the horse metacarpophalangeal joint. Front Vet Sci 10:1045030 (2023). PubMed: 36937015
Paranjape N et al. A CRISPR-engineered isogenic model of the 22q11.2 A-B syndromic deletion. Sci Rep 13:7689 (2023). PubMed: 37169815
Furukawa M et al. Long-Term Soft-Food Rearing in Young Mice Alters Brain Function and Mood-Related Behavior. Nutrients 15:N/A (2023). PubMed: 37242280
Zhao T et al. Macrophages induce gingival destruction via Piezo1-mediated MMPs-degrading collagens in periodontitis. Front Immunol 14:1194662 (2023). PubMed: 37261355
Xiao X et al. Microglia Sirt6 modulates the transcriptional activity of NRF2 to ameliorate high-fat diet-induced obesity. Mol Med 29:108 (2023). PubMed: 37582706
Liu H et al. Galectin-3 as TREM2 upstream factor contributes to lung ischemia-reperfusion injury by regulating macrophage polarization. iScience 26:107496 (2023). PubMed: 37636061
Zamith Cunha R et al. Endocannabinoid System Receptors at the Hip and Stifle Joints of Middle-Aged Dogs: A Novel Target for the Therapeutic Use of Cannabis sativa Extract in Canine Arthropathies. Animals (Basel) 13:N/A (2023). PubMed: 37760233
Tong X et al. Rotator cuff healing is regulated by the lymphatic vasculature. J Orthop Translat 38:65-75 (2023). PubMed: 36313978
Zhao B et al. Catalpol ameliorates CFA-induced inflammatory pain by targeting spinal cord and peripheral inflammation. Front Pharmacol 13:1010483 (2022). PubMed: 36353492
Yin S et al. Engineering 2D Silicene-Based Mesoporous Nanomedicine for In Vivo Near-Infrared-Triggered Analgesia. Adv Sci (Weinh) 9:e2202735 (2022). PubMed: 35750652
Sun H et al. Bacteria reduce flagellin synthesis to evade microglia-astrocyte-driven immunity in the brain. Cell Rep 40:111033 (2022). PubMed: 35793624
Huang S et al. Increased CD34 in pancreatic islet negatively predict islet β-cell decrease in type1 diabetes model. Front Physiol

13:1032774 (2022). PubMed: 36467676
Naz N et al. Cerebral Folate Metabolism in Post-Mortem Alzheimer's Disease Tissues: A Small Cohort Study. Int J Mol Sci 24:N/A (2022). PubMed: 36614107
Chen S et al. Microcrystalline silica particles induce inflammatory response via pyroptosis in primary human respiratory epithelial cells. Environ Toxicol 37:385-400 (2022). PubMed: 34766707
Aranaz-Novaliches G et al. Multi-Level Approach for Comprehensive Enamel Phenotyping. Curr Protoc 2:e340 (2022). PubMed: 35007410
Theocharidis G et al. Single cell transcriptomic landscape of diabetic foot ulcers. Nat Commun 13:181 (2022). PubMed: 35013299
Vankriekelsvenne E et al. Transmembrane protein 119 is neither a specific nor a reliable marker for microglia. Glia 70:1170-1190 (2022).

Animals and other research organisms

Policy information about studies involving animals; ARRIVE guidelines recommended for reporting animal research, and Sex and Gender in Research

Laboratory animals	B6.129P2(Cg)-Cx3cr1 tm1Litt /J (JAX); Sprague Dawley (Crl:CD(SD) Outbred)
Wild animals	The study did not involve wild animals.
Reporting on sex	7 males and 7 females mice were used in the study. 8 male rats were used for electrophysiology.
Field-collected samples	The study did not involve samples collected in the field.
Ethics oversight	Subjects were single housed with environmental enrichment under a 12hr light-dark cycle in a climate-controlled room (20°C ± 2.5°C and relative humidity 30-70%) with continuous access to food and water in accordance with IACUC guidelines. All animal experiments were performed in accordance with the NIH Guide for Care and Use of Animals and approved by the University of Pittsburgh Institutional Animal Care and Use Committee.

Note that full information on the approval of the study protocol must also be provided in the manuscript.

Plants

Seed stocks	*Report on the source of all seed stocks or other plant material used. If applicable, state the seed stock centre and catalogue number. If plant specimens were collected from the field, describe the collection location, date and sampling procedures.*
Novel plant genotypes	*Describe the methods by which all novel plant genotypes were produced. This includes those generated by transgenic approaches, gene editing, chemical/radiation-based mutagenesis and hybridization. For transgenic lines, describe the transformation method, the number of independent lines analyzed and the generation upon which experiments were performed. For gene-edited lines, describe the editor used, the endogenous sequence targeted for editing, the targeting guide RNA sequence (if applicable) and how the editor was applied.*
Authentication	*Describe any authentication procedures for each seed stock used or novel genotype generated. Describe any experiments used to assess the effect of a mutation and, where applicable, how potential secondary effects (e.g. second site T-DNA insertions, mosiacism, off-target gene editing) were examined.*
